# Exploring the role of cyclin D1 in the pathogenesis of multiple myeloma beyond cell cycle regulation

**DOI:** 10.1002/1878-0261.70085

**Published:** 2025-07-17

**Authors:** Ignacio J. Cardona‐Benavides, Sara Cristobal‐Vargas, Cristina De Ramón, Elizabeta A. Rojas, Irena Misiewicz‐Krzeminska, Isabel Isidro, José Juan Pérez, Bruno Paiva, Noemi Puig, Miguel Alcoceba, María‐Victoria Mateos, Luis A. Corchete, Myriam Cuadrado, Norma C. Gutiérrez

**Affiliations:** ^1^ Hematology Department, Institute of Biomedical Research of Salamanca (IBSAL) University Hospital of Salamanca Spain; ^2^ Cancer Research Center‐IBMCC (USAL‐CSIC) Salamanca Spain; ^3^ Department of Experimental Hematology Institute of Hematology and Transfusion Medicine Warsaw Poland; ^4^ Clínica Universidad de Navarra, Centro de Investigaciones Biomédicas Aplicadas (CIMA) Instituto de Investigación Sanitaria de Navarra (IdiSNA) Pamplona Spain; ^5^ Centro de Investigación Biomédica en Red de Cáncer (CIBERONC) Madrid Spain; ^6^ Krantz Family Center for Cancer Research Massachusetts General Hospital Cancer Center Boston MA USA; ^7^ Harvard Medical School Boston MA USA; ^8^ Broad Institute of MIT and Harvard Cambridge MA USA

**Keywords:** adhesion, CD56, CTC, cyclin D1, multiple myeloma

## Abstract

Cyclins D could be a unifying event in multiple myeloma (MM), even though MM is not typically considered a proliferative disease. In this study, we hypothesized that cyclins D might have additional roles in the pathogenesis of MM beyond cell cycle control. We showed that overexpression of *CCND1* and *CCND2* in MM cell lines lacking these proteins revealed a mutually exclusive expression pattern, with both cyclins D localized in the cytoplasm and no impact on proliferation. To investigate non‐canonical roles of cyclin D1, we performed transcriptome analysis and multidimensional flow cytometry. Cyclin D1 overexpression led to upregulation of several key cell adhesion pathway proteins, including STAT1 and ZO‐1, along with alterations in the actin cytoskeleton and decreased adhesion to certain matrices. Immunophenotypic analysis showed a significant reduction in CD56 expression following cyclin D1 overexpression, validated in a cohort of 85 MM patients, in which 73% with high cyclin D1 were CD56‐negative. High cyclin D1 was also associated with increased circulating tumor cells (CTCs) (*P* < 0.001). Overall, we revealed novel functions of cyclin D1 in MM pathogenesis, particularly in cell adhesion and dissemination.

## Graphical abstract

Cyclin D1 overexpression altered the cell adhesion pathway, while cyclin D2 upregulation had less impact on pathway enrichment analysis. Multiple myeloma (MM) patients with cyclin D1 overexpression showed reduced CD56 expression and increased circulating tumor cells (CTC) levels, suggesting that cyclin D1 may contribute to MM cell dissemination capacity.

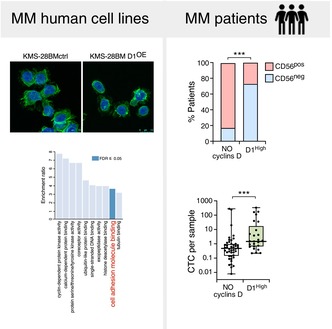

**Abbreviations**
ATCC: American Type Culture Collection
BM: bone marrow
CNIA: capillary electrophoresis nanoimmunoassay
CTC: circulating tumor cell
DAPI: 4′,6‐diamidino‐2‐phenylindole
DSMZ: German Collection of Microorganisms and Cell Cultures
FBS: fetal bovine serum
FC: fold change
FDR: false discovery rate
FLNA: filamin A
JCRB: Japanese Collection of Research Bioresources
KMS‐28BM_D1^OE^
: KMS‐28BM cells overexpressing cyclin D1
KMS‐28BM_D2^OE^
: KMS‐28BM cells overexpressing cyclin D2
KMS‐28PE_D1^OE^
: KMS‐28PE cells overexpressing cyclin D1
KMS‐28PE_D2^OE^
: KMS‐28PE cells overexpressing cyclin D2
MM: multiple myeloma
MTT: 3‐(4,5‐dimethylthiazol‐2‐yl)‐2,5‐diphenyl‐2H‐tetrazolium bromide
OPM2_D1^OE^
: OPM2 cells overexpressing cyclin D1
PC: plasma cell
PE: pleural exudate
PLL: poly‐l‐lysine
RT: room temperature
SD: standard deviation

## 1 Introduction

Multiple myeloma (MM) accounts for 2% of all cancers and approximately 10% of hematological malignancies, making it the second most common blood‐based cancer [1, 2]. It is characterized by an abnormal accumulation of malignant plasma cells (PCs) within the bone marrow (BM) that leads to end‐organ damage, including osteolytic bone lesions, hypercalcemia, renal disease, and anemia [3, 4]. MM is genomically highly heterogeneous at the intertumoral and intratumoral levels [5, 6, 7, 8]. Initial genetic events include translocations involving *IGH* genes, such as t(4;14), t(11;14), t(14;16), and t(14;20), as well as hyperdiploidy. Other key genetic lesions associated with progression include abnormalities on chromosome 1, deletions of 17p, and secondary translocations of the *MYC* gene [5, 9]. Despite this genetic diversity at the DNA level, seminal gene expression profiling studies have found overexpression of *CCND1*, *CCND2*, or *CCND3* mRNA in nearly all MM cases, suggesting that a potential unifying event occurs in MM pathogenesis [10, 11]. Thus, *CCND1* is directly overexpressed due to t(11;14), polysomy 11, or, as shown more recently, by templated insertions; *CCND3* is directly overexpressed due to t(6;14); and high *CCND2* mRNA levels were observed in the remaining MM cases, including those with t(4;14), t(14;16), and in some hyperdiploid and non‐hyperdiploid MM patients [8, 10, 12, 13, 14]. The mechanisms by which *CCND2* was overexpressed in these cases have not been fully elucidated [15, 16, 17, 18]. Nevertheless, our group has recently observed that, at the protein level, not all patients with MM have increased expression of one of the three cyclins D. In fact, no expression of any of the cyclin D proteins was detected in 41% of MM patients [19].

The canonical and widely acknowledged function of the three cyclins D is to control the transition from the G1 to the S phase of the cell cycle [20, 21, 22, 23]. Given the pivotal role of cyclins D in driving cell cycle progression, it is difficult to reconcile the finding that almost all MMs overexpress one of the cyclins D with the fact that MM tumor cells have a low proliferative index. This discrepancy prompts us to hypothesize that D type cyclin involvement in MM pathogenesis may potentially transcend the conventional function of proliferation promotion. To validate this hypothesis, we conducted experiments in which cyclin D1 and cyclin D2 were ectopically overexpressed in several MM cell lines. Remarkably, our findings revealed that cyclin D1 overexpression led to the deregulation of some genes associated with cellular adhesion pathways.

## 2 Materials and methods

### 2.1 MM cell lines and primary patient samples

The human myeloma cell lines KMS12‐BM (RRID:CVCL_1334), KMS12‐PE (RRID:CVCL_1333), AMO‐1 (RRID:CVCL_1806), JJN3 (RRID:CVCL_2078), and OPM2 (RRID:CVCL_1625) were acquired from DMSZ (German Collection of Microorganisms and Cell Cultures), U266 (RRID:CVCL_0566), MM1.S (RRID:CVCL_8792), and HEK293T (RRID:CVCL_0063) cell lines were procured from ATCC (American Type Culture Collection) and KMS‐28BM (RRID:CVCL_2994) and KMS‐28PE (RRID:CVCL_2995) were supplied by JCRB (Japanese Collection of Research Bioresources) Cell Bank. Cytogenetic alterations of each cell line can be found in Table S1. MM cell lines were cultured in RPMI 1640 medium supplemented with 10–20% fetal bovine serum (FBS), while HEK293T cells were grown in 10% FBS‐DMEM medium. Both media were supplemented with penicillin/streptomycin (Gibco Life Technologies, Waltham, MA, USA). Cell line identity was confirmed periodically by STR analysis with the PowerPlex 16 HS system kit (Promega, Madison, Wiscosin, USA) and online STR matching analysis. Mycoplasma contamination was assessed using the MycoAlert^®^ Detection Kit (Lonza Group, Basel, Switzerland).

Samples from patients with newly diagnosed myeloma treated within the framework of the Spanish Myeloma Group clinical trial GEM2012 (NCT01916252) were used [24]. The GEM2012 trial had been approved by the local ethics committee and implemented in agreement with the Declaration of Helsinki. Written informed consent from patients was required before they could participate in the clinical trial.

### 2.2 RNA and protein extraction

RNA and protein extractions were performed using the AllPrep DNA/RNA Mini Kit (Qiagen, Hilden, Germany). Subsequently, proteins were extracted by precipitation in ice‐cold acetone, as previously described by our group [25].

Nucleus/cytoplasm fractions were extracted with NE‐PER™ Nuclear and Cytoplasmic Extraction Reagents (Thermo Fisher, Waltham, MA, USA).

### 2.3 Gene overexpression and lentiviral generation

Overexpression of *CCND1* and *CCND2* genes was achieved using plasmids designed by VectorBuilder™: pLV‐EGFP‐MSCV>hCCND1 [NM_053056.3] and pLV‐EGFP‐MSCV>hCCND2 [NM_001759.4], respectively. Cells were transduced using lentiviral particles. For this purpose, HEK293T cells were transfected at 70% confluency with the required plasmids and the helper lentiviral plasmids (Addgene psPAX2 #12260 and pMD2G #12259, Watertown, MA, USA) using Lipofectamine 2000 (Life Technologies, Carlsbad, CA, USA). The lentiviral supernatant was collected and filtered using 0.45 μm Millipore Stericup filters (Merck, Rahway, NJ, USA) before being used to infect. *CCND*‐overexpressing cells were selected by sorting GFP‐positive cells in a BD FACSAria II cytometer.

### 2.4 Capillary electrophoresis nanoimmunoassay (CNIA)

Capillary electrophoresis nanoimmunoassay (CNIA) was performed in the WES and Abby system (ProteinSimple, San Jose, CA, USA) according to the manufacturer's protocol and the previous experience of our group [25, 26, 27]. The primary antibodies, used under pre‐optimized conditions, were cyclin D1 (Abcam, Cambridge, UK, ab134175, 1/50 dilution), cyclin D2 (Cell Signaling, #3741, 1/50 dilution), cyclin D3 (Abcam, ab52598, 1/100 dilution), β‐tubulin (Cell Signaling, Danvers, MA, USA, #2146, 1/50 dilution), PARP (Cell Signaling, #9542S, 1/50 dilution), ZO‐1 (Proteintech, Manchester, UK, 21773‐1‐AP, 1/200 dilution), STAT1 (Cell Signaling, #14994, 1/50 dilution), FLNA (Cell Signaling, #4762, 1/25 dilution), and GAPDH (Cell Signaling, #2118, 1/50 dilution).

Expression of cyclin D proteins was normalized with respect to GAPDH expression. For subcellular localization experiments, levels of protein expression were reported relative to β‐tubulin and PARP proteins for the cytoplasmic and nuclear fractions, respectively. ZO‐1, STAT1, and FLNA protein expression levels were normalized to the total protein amount.

### 2.5 Quantitative real‐time PCR

RNA concentration and integrity were assessed with NanoDrop™. Using the SuperScript II First‐Strand Synthesis kit (Thermo Fisher), approximately 200 ng of total RNA was reverse‐transcribed into oligo‐dT cDNA. Gene expression of *CCND1* (Hs00765553_m1), *CCND2* (Hs00153380_m1), *CCND3* (Hs05046059_s1), *STAT1* (Hs01013996_m1), *TJP1* (Hs01551871_m1), *FLNA* (Hs00924645_m1), *ICAM1* (Hs00164932_m1), *LCP1* (Hs00158701_m1), and *CNN2* (Hs00854264_s1) was evaluated with TaqMan qRT‐PCR assays (Thermo Fisher). Relative gene expression was calculated by the 2^−ΔCt^ method using PGK1 (Hs00943178_g1) as the housekeeping gene for normalization. Experiments were performed a minimum of three times.

### 2.6 MTT cell proliferation assay

Cells were seeded in 96‐well plates (15 000 cells per well) for 24, 48, and 72 h. Cell proliferation was determined using 3‐(4,5‐dimethylthiazol‐2‐yl)‐2,5‐diphenyl‐2H‐tetrazolium bromide (MTT) (Sigma‐Aldrich, St. Louis, MI, USA). 10 μL of MTT (5 mg·mL^−1^), dissolved in PBS, were added per well. After 2 h incubation, formazan crystals were dissolved in isopropanol with 1% HCl (100 μL per well). Absorbance was measured on a plate reader (TECAN Ultra Evolution, Männedorf, Switzerland), at a wavelength (λ) of 570 nm, using a reference λ of 630 nm. Six wells were analyzed for each condition, and the results are presented as the mean ± standard deviation. Each experiment was conducted a minimum of three times.

### 2.7 EdU cell proliferation assay

Cell cycle was analyzed with Click‐iT™ Plus EdU Alexa Fluor™ 647 Flow Cytometry (Thermo Fisher) according to the manufacturer's protocol. Cells were acquired using a BD AccuriC6+™ flow cytometer, and data were analyzed with flowjo™ software (v10.9) (BD, Franklin Lakes, NJ, USA). Experiments were performed a minimum of three times.

### 2.8 Transcriptome arrays

Total RNA was hybridized on the GeneChip™ Clariom S from Thermo Fisher. Total RNA was quantified, amplified, labeled, and hybridized on the GeneChip™ Clariom S from Thermo Fisher. Signal intensity values from microarray data were obtained from CEL files. Raw data were normalized with the ‘RMA’ algorithm from the ‘oligo’ package v1.60 in R v4.3.2 using the custom BrainArray ENSG CDF file (v25.0.0) for gene annotation. Unsupervised multidimensional scaling (MDS) and hierarchical dendrograms were generated in R. Dissimilarity matrices and hierarchical cluster analyses were calculated using the stats package (v3.6.2), taking the Euclidean distance as the distance measure and the group average method as the linkage method. The associated graphs were created with the FactoMineR (v2.11) and factoextra (v1.0.7) packages. Differentially expressed genes were identified using the significance analysis of microarrays (SAM) method from the ‘siggenes’ package (v1.78.0). Adjusted *P*‐values were calculated by applying the Benjamini–Hochberg correction (false discovery rate, FDR). Genes with an FDR < 0.05 were considered statistically significant, and deregulated genes were identified using a log_2_‐fold change (FC) > 1.5. Analysis of overrepresentation of KEGG pathways and GO functions was performed in Webgestalt 2024. Full microarray data are available from Gene Expression Omnibus under accession number GSE277542.

### 2.9 Cell migration assay

Migration assays were carried out in Transwell^®^ Permeable Supports, with 6.5 mm of insert in 24‐well plates and 5.0 μm polycarbonate membrane (Sarstedt, Nümbrecht, Germany). Cells were starved in serum‐free culture medium for 12 h. A final concentration of 1 × 10^6^ cells per 100 μL per condition was seeded in the upper chamber of the Transwell, and the lower compartment was filled with serum‐free medium or 20% FBS‐RPMI1640. After 24 h of incubation at 37 °C and 5% CO_2_, cells migrating into the lower chambers were collected and counted using a BD AccuriC6+ flow cytometer. All conditions were performed in triplicate, and overexpression and control samples were tested in parallel.

### 2.10 Cell adhesion assay

48‐well plates were coated with 200 μL per well of different substrates and incubated under various conditions (LDEV Geltrex, fibronectin, poly‐l‐lysine, and collagen type‐I) (Table S2). Cell lines were prepared at a final concentration of 5 × 10^4^ cells per 100 μL per condition and added to each well that had previously been blocked with 10% FBS‐RPMI1640 for 30 min at room temperature (RT). Cells were allowed to adhere at 37 °C for different times (1, 4, and 24 h), and non‐adherent cells were removed by washing with 1% BSA‐RPMI, fixed with 4% PFA for 15 min at RT and stained for 10 min with crystal violet (0.5% w/v in 20% ethanol) at RT. The excess crystal violet was then removed by immersion washing in distilled water and the plates were allowed to dry. After drying, 200 μL of 2% SDS/PBS were added and, after incubation in the dark and shaking for 30 min at RT, the absorbance was read on a TECAN (infinite M200 PRO) system at 550 μm. Wells with only the substrate (no cells) were used as the baseline for normalization. Parental and cyclin D‐overexpressing cell lines were analyzed in parallel. Each condition was tested in technical duplicates and biological triplicates.

### 2.11 Immunofluorescence microscopy

Cells in suspension were plated onto poly‐l‐lysine (PLL)‐coated coverslips (10 μg·mL^−1^, Sigma) for 1 h. Afterwards, cells were fixed in 3% PFA for 10 min at 37 °C and permeabilized with 0.5% Triton X‐100 for 10 min at RT, washed twice with PBS, blocked in 2% BSA in PBS for 30 min, and then incubated in a wet chamber with primary antibodies followed by the secondary antibody. Finally, cells were stained with 1 μg·mL^−1^ of 4′,6‐diamidino‐2‐phenylindole (DAPI; Invitrogen) for 3 min to visualize the nuclei. Images were captured with a Laser Confocal Microscopy Leica SP5.

### 2.12 Next‐generation flow cytometry

Next‐generation flow cytometry in bone marrow and peripheral blood samples was conducted according to the method developed by EuroFlow for MM [28], which reaches an estimated sensitivity of 2 × 10^−6^. In brief, 3–5 mL of bone marrow and 5–8 mL of peripheral blood were bulk‐lysed with ammonium chloride, and then, ≥ 10 × 10^6^ cells were stained with the eight‐color, two‐tube antibody panel for accurate identification, quantification, and characterization of phenotypically aberrant, clonal plasma cells (PCs): tube 1 includes CD138‐BV421, CD27‐BV510, CD38‐FITC, CD56‐PE, CD45‐PerCPCy5.5, CD19‐PECy7, CD117‐APC, and CD81‐APCH7; tube 2 includes CD138‐BV421, CD27‐BV510, CD38‐FITC, CD56‐PE, CD45‐PerCPCy5.5, CD19‐PECy7, cyKAPPA‐APC, and cyLAMBDA‐APCH7 [29]. The immunophenotype of MM cell lines was characterized using only tube 1. The methodology for defining and quantifying CTCs, as well as all technical and analytical procedures, is fully detailed in the previously published study [30]. In the present manuscript, we focused on comparing the number of CTCs between two specific groups of patients: those with no detectable expression of cyclin D1 and D2 protein, and those with high expression levels of cyclin D1. The methodology for CTC quantification was not altered for this analysis. Data were acquired with an FACSCanto II flow cytometer (BD) using the facsdiva 6.1 software (BD) and analyzed by infinicyt software (Cytognos SL, Salamanca, Spain).

### 2.13 Statistical analysis

The statistical significance of group differences was assessed using a two‐tailed unpaired Student's *t*‐test. Variance homogeneity was evaluated with the *F* test, and if this assumption was not satisfied, Welch's *t*‐test was applied instead of Student's *t*‐test. Data normality was checked using the Shapiro–Wilk test. Outliers were identified and excluded from further analysis by the ROUT method. The Mann–Whitney *U*‐test was used when variables did not meet the *t*‐test assumptions. The relationship between categorical variables was analyzed using Fisher's exact test. The data analysis and graphical representations were performed using graphpad prism 10.2.1 software (Boston, MA, USA). All experiments were replicated at least three times. The error bars in the figures indicate the SD. Differences were considered statistically significant for values of *P* < 0.05 (*) and highly significant for values of *P* < 0.01 (**) and *P* < 0.001 (***).

## 3 Results

### 3.1 Expression levels and subcellular localization of cyclin D proteins in MM cell lines

**Fig. 1:**
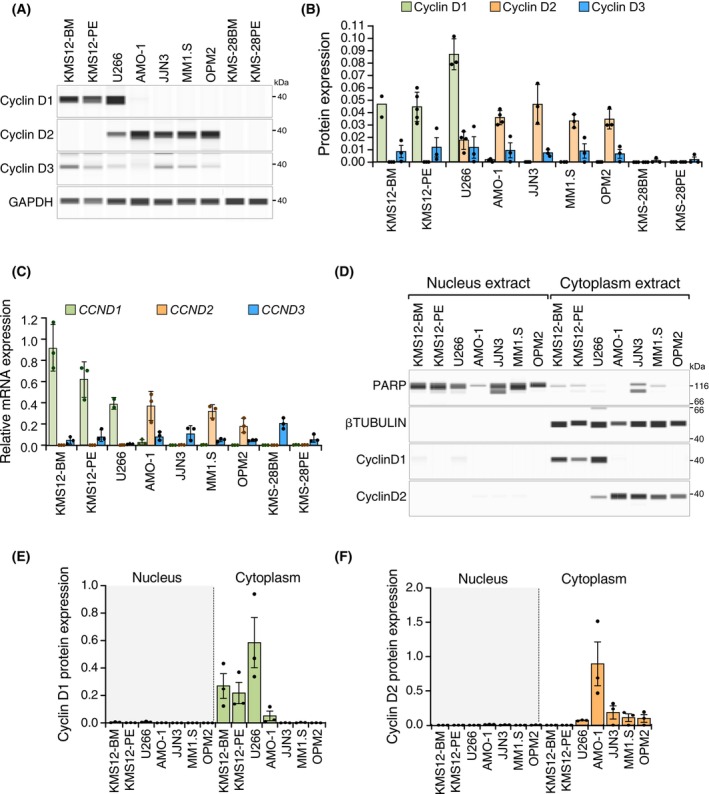
Basal cyclin D1 and cyclin D2 expression in multiple myeloma (MM) cell lines. (A) Basal protein expression levels of cyclin D1, cyclin D2, cyclin D3, and GAPDH measured by CNIA technology in MM cell lines. (B) Quantification of basal protein expression levels of cyclin D1, cyclin D2, and cyclin D3 in three independent experiments. Data were normalized with respect to GAPDH protein expression. (C) Quantification of basal mRNA expression levels of *CCND1*, *CCND2*, and *CCND3* normalized with respect to *PGK1*. (D) Basal expression of cyclin D1, cyclin D2, PARP, and β‐tubulin proteins measured by CNIA technology in nucleus and cytoplasm extracts of MM cell lines. (E) Quantification of basal cyclin D1 protein expression levels in nucleus and cytoplasm extracts. (F) Quantification of basal cyclin D2 protein expression in nucleus and cytoplasm extracts. Error bars indicate the SD. In panels (E) and (F) data were normalized with respect to PARP and β‐tubulin expression for the nucleus and the cytoplasm, respectively. Data are from three independent experiments.

We assessed the mRNA and protein expression level of cyclins D1 and D2 by qRT‐PCR and CNIA, respectively, in nine MM cell lines (Fig. 1). Most of these cell lines exhibited exclusive expression of either cyclin D1 or cyclin D2 at the mRNA and protein levels. Specifically, the KMS12‐BM and KMS12‐PE cell lines exclusively expressed cyclin D1 protein, whereas the AMO‐1, JJN3, MM1.S, and OPM2 cell lines expressed only cyclin D2 protein. The U266 cell line co‐expressed both cyclin D types, albeit with a preference for cyclin D1. Notably, the KMS‐28BM and KMS‐28PE cell lines did not express either cyclin D1 or cyclin D2 (Fig. 1A–C). Regarding cyclin D3, protein expression levels were low across all MM cell lines analyzed, except for KMS‐28BM and KMS‐28PE, where it was not detectable.

Next, we analyzed the subcellular compartment in which cyclin D was concentrated. To this end, we performed a cell fractionation assay to discriminate between the nucleus and cytoplasm, and both fractions were tested for cyclin D protein levels in the nine aforementioned cell lines. This analysis showed that both cyclins were preferentially present in the cytoplasm of all the cell lines analyzed (Fig. 1D–F). We also observed in the U266 cell line, by immunofluorescence, the cytoplasmic preference for both cyclin D1 and D2 (Fig. S1A,B).

### 3.2 Effect of cyclin D1 or cyclin D2 overexpression in MM cell lines

**Fig. 2:**
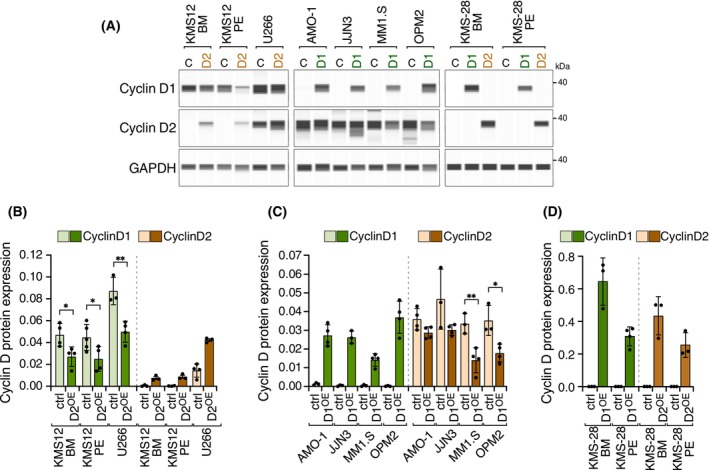
Effects of ectopic cyclin D overexpression on endogenous cyclin D1 and cyclin D2 levels in multiple myeloma (MM) cell lines. (A) Cyclin D1, cyclin D2, and GAPDH protein expression measured by CNIA technology in MM lines. Cyclin D2 (brown) was overexpressed in KMS12‐BM, KMS12‐PE, and U266 cell lines. Cyclin D1 (green) was overexpressed in AMO‐1, JJN3, MM1.S, and OPM2 cell lines. Cyclin D1 and cyclin D2 were both overexpressed in KMS‐28BM and KMS‐28PE. (B) Quantification of cyclin D1 and D2 protein expression in cell lines with exogenous overexpression of cyclin D2 (D2^OE^). Basal levels are shown in light green (cyclin D1) and light brown (cyclin D2), while levels after cyclin D2 overexpression are shown in dark green and dark brown, respectively. (C) Quantification of cyclin D1 and cyclin D2 protein expression in cell lines with exogenous overexpression of cyclin D1 (D1^OE^). Basal levels are shown in light green (cyclin D1) and light brown (cyclin D2), while levels after cyclin D1 overexpression are shown in dark green and dark brown, respectively. (D) Quantification of cyclin D1 and cyclin D2 protein expression in KMS‐28BM and KMS‐28PE cell lines. Basal levels are shown in light green (cyclin D1) and light brown (cyclin D2), while levels after overexpression of cyclin D1 and cyclin D2 are shown in dark green and dark brown, respectively. Error bars indicate the SD. For quantification, data were normalized with respect to GAPDH protein expression. Data are from three independent experiments. An unpaired *t*‐test was used to establish the significance of group differences (*P < 0.05, **P < 0.01), except for the JJN3 cell line, for which Welch's *t*‐test was applied because of unequal variances.

**Fig. 3:**
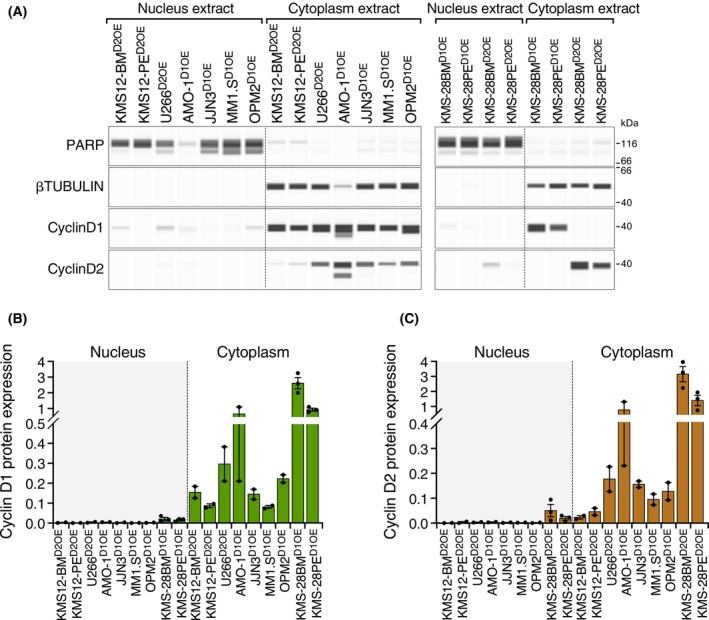
Subcellular localization of cyclin D proteins in multiple myeloma (MM) cell lines after ectopic cyclin D overexpression. (A) Expression of cyclin D1, cyclin D2, PARP, and β‐tubulin proteins measured by CNIA technology in nuclear and cytoplasmic extracts of cyclin D‐overexpressing MM cell lines. (B) Quantification of cyclin D1 protein expression in nuclear and cytoplasmic extracts from cyclin D‐overexpressing MM cell lines. (C) Quantification of cyclin D2 protein expression in nuclear and cytoplasmic extracts from cyclin D‐overexpressing MM cell lines. Error bars indicate the SD. In panels (B) and (C), data were normalized with respect to PARP and β‐tubulin protein expression for nucleus and cytoplasm, respectively. Data represents three independent experiments.

Cyclins D are generally expressed in a mutually exclusive manner [8, 10, 11], so it is reasonable to hypothesize that increasing the expression levels of one cyclin D could lead to a decrease in that of the other. To investigate this possibility, cyclins D1 and D2 were overexpressed in the respective cell lines that did not express this protein. Moreover, cyclin D1 and cyclin D2 were independently overexpressed in the KMS‐28BM and KMS‐28PE cell lines, which did not express either cyclin D. The overexpression of mRNA and protein was checked by qRT‐PCR (Fig. S2) and CNIA (Fig. 2A–D), respectively. Ectopic overexpression of cyclin D1 or cyclin D2 led to a significant reduction in endogenous levels of the alternative cyclin D protein in most cases (Fig. 2B,C), except in the AMO‐1 and JJN3 cell lines. Notably, no changes in the endogenous mRNA levels of cyclins D were seen (Fig. S2). Similar to what was observed in the parental cell lines, the ectopic overexpression of cyclin D1 or cyclin D2 was localized in the cytoplasm (Fig. 3A–C).

Overexpression of cyclin D, given its known role in cell cycle control, has been shown to increase the proliferation rate in other tumors [31, 32, 33]. To test whether the same occurs in MM cell lines, we analyzed proliferation by MTT and quantified cell cycle stages by EdU assay. We found that overexpression of either cyclin D failed to increase the proliferation rate of the MM cell lines (Figs S3 and S4). This, and the observation that cyclins D in myeloma cells localize in the cell cytoplasm, led us to speculate that the overexpression of cyclin D in the MM cells could mediate functions other than those of cell cycle control.

### 3.3 Gene expression profiling of KMS‐28BM and KMS‐28PE cell lines with cyclin D1 or D2 overexpression

To investigate the previous hypothesis and uncover additional roles of cyclins D in myeloma cells, we explored the gene expression changes induced by cyclin D overexpression in KMS‐28BM and KMS‐28PE cell lines, which lack endogenous cyclin D expression.

**Fig. 4:**
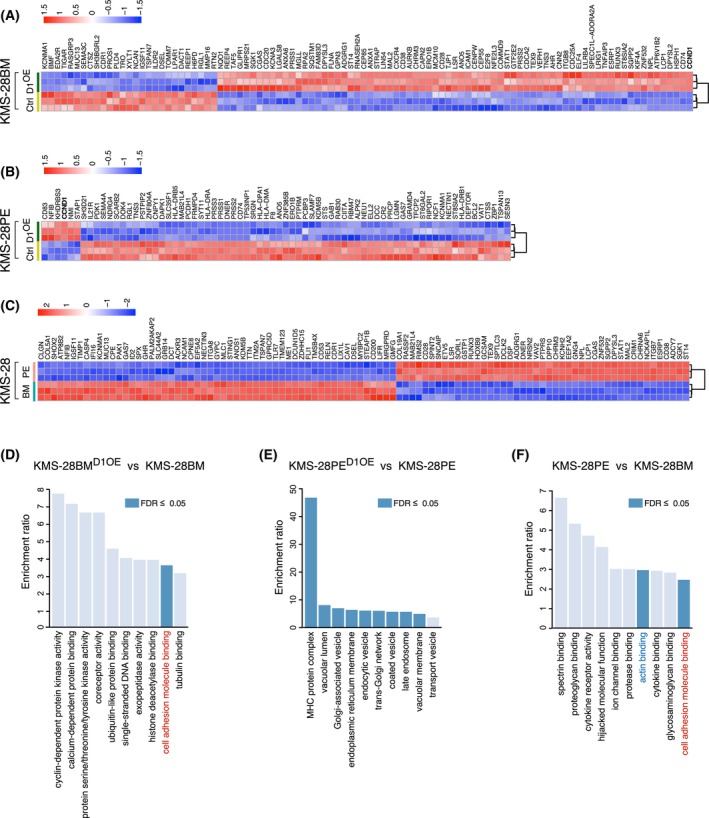
Gene expression profiling and pathway enrichment analysis in cyclin D‐overexpressing multiple myeloma (MM) cell lines. Heatmap displaying the top 100 most deregulated genes that met the defining criteria of > 1.5 log2 fold change (FC) and *P* < 0.05 in (A) KMS‐28BM_D1^OE^ compared with KMS‐28BM cells, (B) KMS‐28PE_D1^OE^ compared with KMS‐28PE cells, and (C) KMS‐28BM and KMS‐28PE parental cell lines. Molecular function pathway enrichment analysis for the comparisons: (D) KMS‐28BM_D1^OE^ vs. KMS‐28BM, (E) KMS‐28PE_D1^OE^ vs. KMS‐28PE, and (F) KMS‐28BM vs. KMS‐28PE. The Webgestalt 2024 suite was used for the enrichment analysis. FDR, false discovery rate.

Comparing the gene expression profile of KMS‐28BM cell line overexpressing cyclin D1 (KMS‐28BM_D1^OE^) and that of the parental KMS‐28BM cells, we identified 115 differentially expressed genes (Fig. 4A). The analysis of deregulated pathways revealed several significantly altered biological process pathways, in particular the regulation of cell adhesion (Fig. S5A). Regarding molecular functions, the cell adhesion molecule binding pathway was the only significantly enriched pathway (Fig. 4D). Analysis of the differential expression of the KMS‐28BM‐overexpressing cyclin D2 (KMS‐28BM_D2^OE^) and KMS‐28BM showed 232 deregulated genes (Fig. S5D), but no significantly altered pathways were detected.

Comparing KMS‐28PE cells overexpressing cyclin D1 (KMS‐28PE_D1^OE^) vs. parental KMS‐28PE (Fig. 4B) revealed 86 differentially expressed genes, while comparison of KMS‐28PE cells overexpressing cyclin D2 (KMS‐28PE_D2^OE^) vs. parental KMS‐28PE (Fig. S5E) identified 60 differentially expressed genes. The analysis of deregulated pathways showed that both KMS‐28PE_D1^OE^ and KMS‐28PE_D2^OE^ exhibited enrichment in antigen processing and presentation pathways, such as the MHC protein complex (Fig. 4E and Fig. S5B,F,G). Specifically, the KMS‐28PE_D1^OE^ cells had notable disruptions in vesicle transport (Fig. 4E). The KMS‐28BM and KMS‐28PE cell lines originated in the same patient, deriving from BM and pleural exudate (PE), respectively. We explored whether their origin could influence the observed expression changes. The comparison between KMS‐28BM and KMS‐28PE revealed 251 differentially expressed genes (the top 100 of these genes are listed in Fig. 4C). The analysis of molecular functions highlighted two significantly enriched pathways: actin binding and cell adhesion molecule binding (Fig. 4F). Notably, the cell adhesion molecule binding pathway was significantly enriched in KMS‐28PE and KMS‐28BM_D1^OE^ relative to KMS‐28BM.

### 3.4 Validation of genes related to the cell adhesion molecule binding pathway

We selected seven upregulated genes, *FLNA*, *CNN2*, *LCP1*, *ICAM1*, *TJP1*, *LGALS8*, and *STAT1*, included in the cell adhesion molecule binding pathway for further investigation. qRT‐PCR was used for validation across the six MM cell lines with cyclin D1 overexpression, and results confirmed consistent patterns of gene upregulation. Specifically, *LCP1*, *ICAM1*, *TJP1*, and *FLNA* genes were upregulated in the KMS‐28BM_D1^OE^ cell line (Fig. S6). Next, we used CNIA to quantify the protein levels of ZO‐1 (*TJP1* gene), STAT1, and FLNA (Fig. S7). STAT1 protein expression was stable across all cases, but ZO‐1 and FLNA expression increased in the KMS‐28BM_D1^OE^ relative to the parental cell line.

**Fig. 5:**
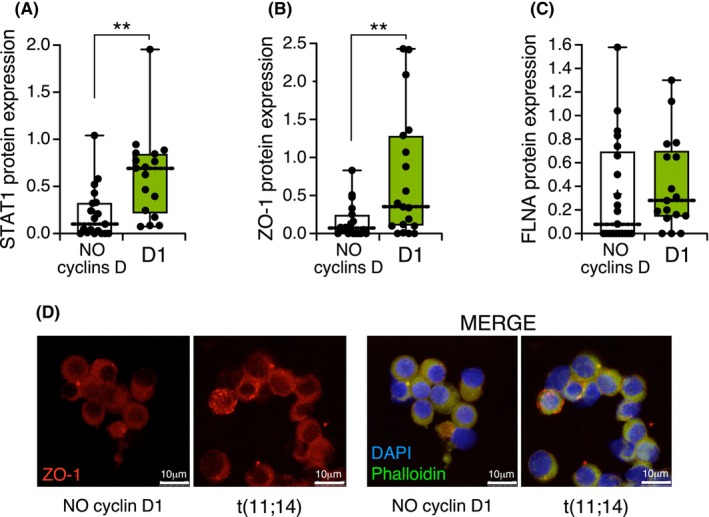
Protein level quantification of STAT1, ZO‐1, and FLNA in MM patients. (A) STAT1, (B) ZO‐1, and (C) FLNA protein expression levels, in patients with cyclin D1 expression (*n* = 17) and those lacking cyclin D expression (*n* = 19). A Mann–Whitney *U*‐test was used to establish the significance of group differences (**P < 0.01). (D) Immunofluorescence staining of ZO‐1 in a patient with t(11;14) compared with a patient lacking cyclin D1 expression. Scale bars are shown in each image and represent 10 μm.

To find out whether these *in vitro* findings could be replicated in MM patient samples, we investigated whether MM patients with cyclin D1 overexpression also exhibited significantly higher protein levels of ZO‐1, STAT1, and FLNA compared to patients without detectable cyclin D protein expression. To address this, we took advantage of our previous study [19] in which information about cyclin D protein expression was available from a large set of MM samples. We assembled two groups of patients: the first comprising 17 patients with the t(11;14) translocation and characterized by elevated cyclin D1 protein expression, and the second comprising 19 patients without detectable cyclin D1 or D2 protein, regardless of their cytogenetic profile. Patients with high cyclin D1 protein expression displayed significantly higher levels of STAT1 (Fig. 5A) and ZO‐1 (Fig. 5B) compared to those in the second group. However, FLNA expression (Fig. 5C) did not differ significantly between the two groups. In the case of ZO‐1, protein localization differed between patients with t(11;14) and those lacking cyclin D1 expression, as verified by immunofluorescence (Fig. 5D). In t(11;14) patients, ZO‐1 formed distinct accumulations, while in cyclin D1‐negative patients, it showed a weaker and more diffuse membrane distribution.

### 3.5 Cyclin D1 overexpression alters cell morphology and reduces adhesion in MM cell lines

**Fig. 6:**
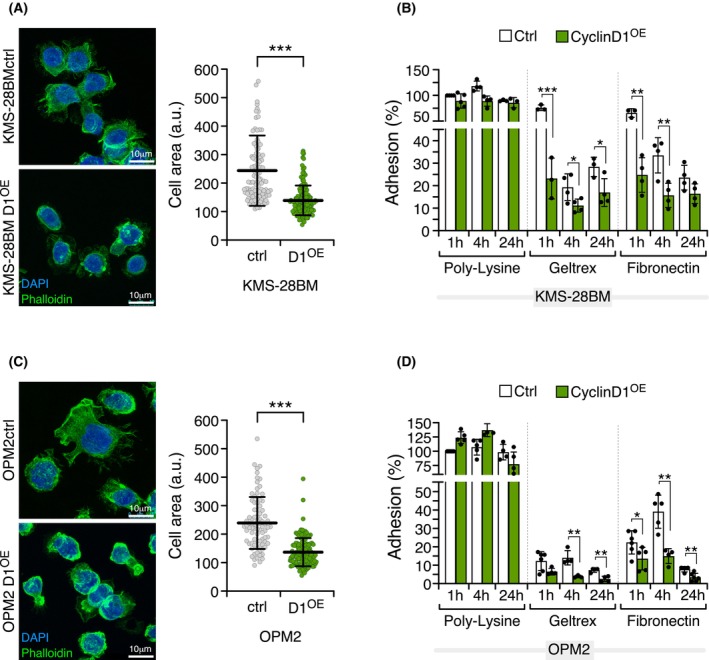
Cell morphology and cell adhesion analysis. Immunofluorescence and quantitative analysis of cell area spreading on poly‐l‐lysine (PLL)‐coated surfaces of (A) KMS‐28BM and KMS‐28BM_D1^OE^, and (C) OPM2 and OPM2_D1^OE^ lines. Parental cell lines are shown in white, and cyclin D1‐overexpressing cell lines are shown in dark green. A Mann–Whitney *U*‐test was used to establish the significance of group differences. (B) and (D) Adhesion analysis on different substrates (PLL, Geltrex, fibronectin) of KMS‐28BM and KMS‐28BM_D1^OE^, and OPM2 and OPM2_D1^OE^ cell lines, respectively. Data are from at least three independent experiments, and the analysis was performed at 1, 4 and 24 h. For normalization, the 1 h PLL time was used for each cell line. Error bars indicate the SD. An unpaired *t*‐test was used to establish the significance of group differences (*P < 0.05, **P < 0.01, ***P < 0.001). Scale bars are shown in each image and represent 10 μm.

The above results led us to hypothesize that cyclin D1 overexpression could induce modifications, or play a role, in the adhesion pathway. To test this, we studied the configuration of the F‐actin cytoskeleton in KMS‐28BM and OPM2 control cells compared with cyclin D1‐overexpressing counterparts. Interestingly, KMS‐28BM_D1^OE^ and OPM2_D1^OE^ cells both displayed alterations in their morphological configuration upon adhesion to a PLL surface (Fig. 6A,C). The quantification of the cell area revealed that the cyclin D1‐overexpressing cells were significantly smaller than the parental cells (Fig. 6A,C). Next, we conducted an adhesion study to determine how cyclin D1‐overexpressing cells adhered to different matrices. We found that KMS‐28BM_D1^OE^ exhibited significantly reduced adhesion to Geltrex and fibronectin matrices compared with the parental KMS‐28BM cell line (Fig. 6B). This finding was corroborated in OPM2_D1^OE^, which showed significantly lower binding to Geltrex and fibronectin compared with the OPM2 cell line (Fig. 6D). After identifying the influence of cyclin D1 overexpression on the actin cytoskeleton's organization and the decrease cell adhesion, we performed a Transwell migration assay to assess potential changes in cell motility dictated by cyclin D1 overexpression. Remarkably, no notable differences were observed between the control and cyclin D1‐overexpressing cells in this assay (Fig. S8). Our data therefore indicate that cyclin D1 overexpression in KMS‐28BM and OPM2 myeloma cell lines leads to changes in cell morphology and reduces cell adhesion ability. However, it does not affect their migration capabilities, at least under our *in vitro* experimental conditions.

### 3.6 Cyclin D1 overexpression generates immunophenotype changes

**Fig. 7:**
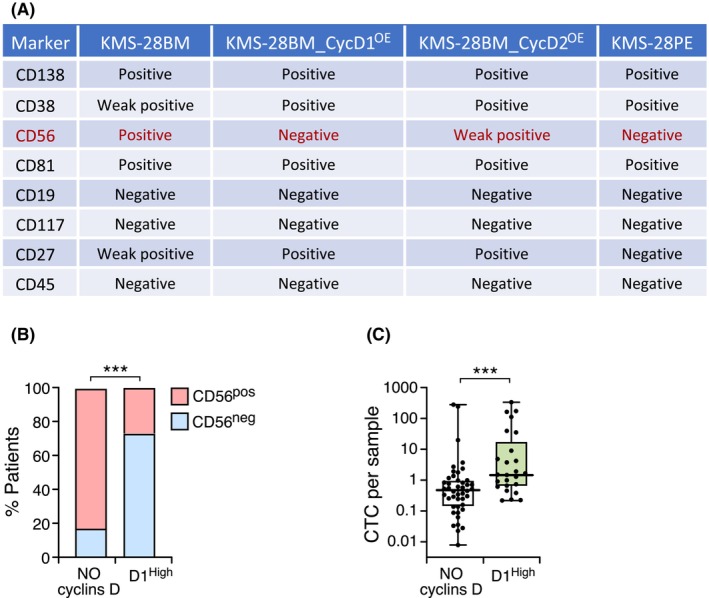
Analysis of immunophenotype and circulating tumor cells (CTCs) in patients with high levels of cyclin D1 expression and no cyclin D expression. (A) Summary table of immunophenotypic markers of MM cell lines. The CD56 marker is highlighted in red, indicating the changes between the KMS‐28BM and KMS‐28BM_D1^OE^ cell lines. (B) Distribution of CD56‐positive and CD56‐negative cells in patients with high (*n* = 22) and no (*n* = 63) cyclin D expression. Data are presented as a contingency table, and Fisher's exact test was used to establish significant proportional differences. (C) Number of CTCs per patient in the comparison between patients with high levels of cyclin D1 expression (*n* = 24) and those lacking cyclin D expression (*n* = 44). Error bars indicate the SD. A Mann–Whitney *U*‐test was used to establish the significance of group differences (***P < 0.001).

We conducted a flow cytometry assay to evaluate key MM markers and determine whether overexpression of cyclin D1 or cyclin D2 in the KMS‐28BM and KMS‐28PE cell lines would also induce changes in their immunophenotype. Our analysis revealed a significant immunophenotypic change in the KMS‐28BM cell line due to cyclin D overexpression. Specifically, the CD38 marker transitioned from weak in the KMS‐28BM line to strong in the KMS‐28BM_D1^OE^ and KMS‐28BM_D2^OE^ cell lines, where it resembled the immunophenotype of the KMS‐28PE cell line. Additionally, the CD56 marker changed, shifting from positive in the KMS‐28BM cell line to weak in the KMS‐28BM_D2^OE^ cell line, and negative in the KMS‐28BM_D1^OE^ line, similar to the KMS‐28PE cell line, which was also negative for this marker (Fig. 7A).

Based on published findings indicating that the CD56 cell adhesion molecule is typically absent from patients with the t(11;14) translocation [34], we investigated CD56 levels in a cohort of 85 MM patients from the GEM2012 trial. Specifically, we explored the association between CD56 expression and cyclin D1 protein overexpression. We categorized MM patients into high cyclin D1 and no cyclins D expression groups and measured CD56 expression in both. The gating strategy and fluorescence intensity profiles for the markers used are presented in Fig. S9. Of the 22 patients with a high level of cyclin D1 expression, 73% (16/22) had CD56‐negative cells, while 27% (6/22) had CD56‐positive cells. In contrast, of the 63 patients with no cyclins D expression, only 17% (11/63) were CD56‐negative, with 83% (52/63) showing CD56 expression (Fig. 7B). The differences between the two groups were significant, supporting the association between high cyclin D1 expression and reduced expression of the CD56 marker.

Since we observed that overexpression of cyclin D1 in the KMS‐28BM cell line led to reduced cell adhesion and loss of the CD56 marker, we explored whether cyclin D1 overexpression was correlated with increased levels of circulating tumor cells (CTCs) in a cohort of MM patients from the GEM2012 trial [30]. For this analysis, we compared 24 patients with elevated cyclin D1 levels with 44 patients lacking detectable levels of cyclin D1 or D2 protein. We found that patients who expressed high levels of cyclin D1 had significantly higher levels of CTCs than those with no expression of cyclins D (Fig. 7C).

## 4 Discussion

The central role of cyclins D in the pathogenesis of MM was established when gene expression studies found that almost all MMs expressed elevated levels of cyclin D‐encoding genes. The established and widely recognized function of cyclins D is to regulate the cell cycle transition from the G1 to the S phase [20, 21, 22, 23]. However, MM stands out as a fundamentally nonproliferative disease characterized by a low division rate. Indeed, our data showed that ectopic overexpression of cyclin D1 or D2 in nine human MM cell lines did not significantly increase their proliferation rate or generate changes in cell cycle stages. These data suggest that cyclin D functions in MM extend beyond proliferation promotion, prompting us to investigate this possibility further.

Cyclin D1 is an oncogene that influences cell growth and genomic stability [35], but also possesses non‐canonical functions when localized in the cytoplasm or bound to the cell membrane. For instance, cyclin D1 can activate the unfolded protein response pathway [36] and inhibit mitochondrial activity in MM, B cells, and other tumors [37]. Moreover, cyclin D1 has been linked to oxidative stress in MM cells, through reactive oxygen species production. This oxidative stress appears to enhance cell migration and adhesion through interactions with actin fibers [38]. In our study, we observed that cyclin D proteins, both endogenous and ectopically overexpressed, are predominantly localized in the cytoplasm of myeloma cells, suggesting that this localization may play a key role in their non‐canonical functions. Attempts to downregulate cyclin D1 or cyclin D2 were unsuccessful, as cells stopped growing after CRISPR‐Cas9 knockout or shRNA knockdown (data not shown). Therefore, we investigated the impact of cyclin D1 and cyclin D2 overexpression on the gene expression profile within the cell lines lacking basal expression of both cyclins D (KMS‐28BM and KMS‐28PE). Cyclin D3 was not included in the current study due to its infrequent overexpression in MM patients and its weak expressions in the MM cell lines analyzed. The pathway enrichment analysis of deregulated genes revealed significant enrichment in the cell adhesion molecule binding pathway in the KMS‐28BM_D1^OE^ line and in the KMS‐28PE cell line (derived from pleural exudate) compared with the KMS‐28BM parental cell line (bone marrow) from the same patient. These findings are consistent with previous reports on mantle cell lymphoma, where cyclin D1 overexpression was associated with cytoplasmic accumulation, contributing to functions beyond cell cycle regulation, such as control of cell migration [39, 40].

Next, several actin‐binding pathway‐related genes were successfully validated in MM cell lines and patient samples. In particular, the expression levels of STAT1, ZO‐1, and FLNA proteins were assessed in a cohort of MM patients, in whom we observed that patients with high levels of cyclin D1 expression exhibited stronger expression of STAT1 and ZO‐1 compared with patients with no expression of cyclins D. In alignment with this result, it has been reported that depletion of STAT1 results in enhanced cell adhesion, suggesting that STAT1 activation reduces adhesion [41]. To better understand the functional implications of this genetic change, we conducted *in vitro* adhesion and migration assays. Our observations revealed that KMS‐28BM and OPM2 cell lines overexpressing cyclin D1 exhibited alterations in their ability to adhere to the analyzed matrix and also displayed a significant change in their spreading pattern on a PLL‐coated surface. Cells with well‐spread morphologies and organized actin stress fibers typically indicate strong adhesion [42]. Specifically, cells overexpressing cyclin D1 retained a rounded morphology and had a more contracted actin cytoskeleton compared with parental cells when they adhered to a PLL‐coated surface. This finding is consistent with a previous study showing an increased detachment capacity of keratinocytes promoted by cytoplasmic cyclin D1 [43]. It has also been reported that cyclin D1 deficiency in macrophages increases adhesion to the matrix and the number of focal points [44].

The overexpression of cyclins D, particularly cyclin D1, in the KMS‐28BM cell line induced significant changes in the immunophenotype, especially in the expression of CD56 marker. This alteration mirrored the immunophenotype observed in the KMS‐28PE cell line. CD56 marker is present in approximately 70% of MM cases [34, 45], and its absence has been associated with the t(11;14) translocation [34]. We confirmed this finding, as we found that patients with high cyclin D1 expression had fewer CD56^+^ cells than those with no cyclin D expression. Notably, the association between reduced CD56 expression and elevated cyclin D1 levels have been functionally demonstrated for the first time *in vitro* by the ectopic overexpression of cyclin D1 in the KMS‐28BM cell line, which resulted in a reduction in CD56 expression.

Investigating the association between cyclin D1 expression and CTC levels could provide valuable insights about the role of this cyclin in MM spreading. We found that patients with high cyclin D1 expression had significantly higher CTC levels than those without cyclin D expression. In conclusion, our results demonstrate that cyclin D1 overexpression in MM patients is associated with the loss of CD56 expression and an increase in CTC levels. Based on these findings, it is reasonable to infer that cyclin D1 overexpression may enhance tumor cell mobility in the bloodstream, potentially explaining why plasma cell leukemias are usually associated with the t(11;14) translocation [46, 47].

In this regard, our Transwell assay findings, which showed no detectable difference between cyclin D1 overexpression and parental cell lines, should be interpreted with caution. This lack of difference could be attributed to the limitations of the *in vitro* assay, which may fail to capture the complex interactions of PCs within the BM microenvironment. It is also possible that cyclin D1 overexpression may contribute to the cell invasion capacity rather than to motility, since cyclin D1 has been shown to phosphorylate a subpopulation of paxillin present in membrane ruffles, promoting Rac1 activation and triggering membrane ruffling and cell invasion [48]. In this context, STAT1 plays a significant role in the migration and invasion mechanisms in various cancer cell types. Recent findings have revealed its involvement in collective invasion, notably in skin cancer cells with high metastatic potential, where the interferon‐β/STAT1 axis serves as the driving force [49]. Overall, collective cell migration involves the coordinated movement of a group of cells and is an essential process with critical roles in embryonic development, wound healing, and cancer metastasis. Furthermore, ZO‐1 is also emerging as a factor involved in collective invasion processes, as cells lacking ZO proteins display reduced collective behavior and impaired mechanical coupling [50]. Notably, both STAT1 and ZO‐1 are upregulated in MM patients with high cyclin D1 expression levels, a condition we have demonstrated to be associated with an increase in CTCs.

Finally, given the similarity of the three cyclin D proteins in structure and function in the cell cycle, they could be assumed to have very similar roles and to be functionally interchangeable. The present study reveals that cyclins D1 and D2 proteins are rarely co‐expressed in MM cell lines, suggesting a pattern of mutual exclusivity between them. This is consistent with previous reports indicating that simultaneous overexpression of *CCND1* and *CCND2* is infrequent in MM patients [10, 51]. Notably, we observed that overexpression of one cyclin D reduces the endogenous levels of the other, supporting the concept of reciprocal exclusivity between cyclins D [8, 10, 11, 52]. While the mechanism underlying this exclusivity remains unknown, one of the few studies addressing this question suggests that cyclin D1 may downregulate *CCND2* expression through modulation of the polyadenylation process [18]. Additionally, our results show that most of the changes in gene expression profiles and molecular pathways are due to cyclin D1 overexpression, with cyclin D2 overexpression introducing fewer changes in the analyzed cell lines. In particular, cyclin D1 overexpression in KMS‐28BM line predominantly influenced gene expression related to cell adhesion pathways, whereas cyclin D2 upregulation had less impact on pathway enrichment analysis. These data raise the possibility that biologically important activities could depend on the cyclin D type. Further experimental validation would be required to confirm this hypothesis.

## 5 Conclusions

In summary, our findings deepen our knowledge of the role of cyclin D proteins in MM biology beyond cell cycle regulation. Specifically, our study highlights novel functions of cyclin D1 in cell adhesion and morphology of myeloma cells, which may influence their dissemination capacity. These insights not only advance our understanding of MM biology but also suggest potential avenues for targeted therapeutic interventions that aim to disrupt cyclin D1‐mediated pathways in MM progression.

## Conflict of interest

CDR receives honoraria from AbbVie and Janssen and travel accommodation from Beigene. BP reports receiving grants and personal fees from BMS, GSK, Sanofi, Takeda, Roche, grants from BeiGene, and personal fees from Janssen, Adaptive, Amgen, and Becton Dickinson. NP reports receiving grants, personal fees, and other support from Amgen, BMS, Pfizer, Janssen, and Takeda; and personal fees from The Binding Site and Sanofi. M‐VM served on speaker bureaus and advisory boards for AbbVie, Adaptive, Amgen, Celgene, GlaxoSmithKline, Janssen, Mundipharma, Oncopeptides, PharmaMar, Roche, Seattle Genetics, and Takeda; NCG receives honoraria from Janssen, Amgen and Sanofi. All the other authors declare no competing financial interests. No related to the present work.

## Author contributions

IJC‐B developed and performed the laboratory experiments, analyzed data, helped prepare the figures, and wrote the manuscript; SC‐V assisted with qRT‐PCR experiments; CDR obtained the patients' clinical data; EAR assisted with CNIA experiments; IM‐K conceived the idea; II performed CNIA experiments and analyzed protein data; JJP, BP, and NP provided and analyzed the flow cytometry data; MA assisted with STR analysis for cell line authentication; LAC analyzed the clinical data and supervised the statistical analysis, helped prepare the figures, and supervised the entire study; M‐VM provided patient samples and clinical data and was responsible for obtaining informed consent from patients; MC assisted with laboratory experiments, analyzed the clinical data, supervised the statistical analysis, wrote the manuscript, prepared the figures, and supervised the whole study; NCG conceived the idea and designed the study, participated in writing the manuscript, supervised the entire study, and provided funding. All authors critically reviewed and approved the manuscript.

## Peer review

The peer review history for this article is available at https://www.webofscience.com/api/gateway/wos/peer‐review/10.1002/1878‐0261.70085.

## Supporting information


**Fig. S1.** Immunofluorescence of cyclins D in the U266 cell line.
**Fig. S2.** Quantification of mRNA expression levels of *CCND1* and *CCND2* in control and overexpressing MM cell lines.
**Fig. S3.** Cell proliferation analysis in multiple myeloma cell lines overexpressing cyclin D1 and cyclin D2.
**Fig. S4.** Cell cycle stage analysis in cyclin D1‐ and cyclin D2‐overexpressing cell lines.
**Fig. S5.** Differential gene expression analysis.
**Fig. S6.** Validation of the expression of genes involved in the cell adhesion molecule binding pathway.
**Fig. S7.** Quantification of STAT1, ZO‐1, and FLNA protein levels in KMS‐28BM and KMS‐28PE cell lines.
**Fig. S8.** Migration analysis of cyclin D1‐overexpressing MM cell lines.
**Fig. S9.** Gating strategy and fluorescence intensity profile of several markers in a CD56‐positive and CD56‐negative MM patient.
**Table S1.** Cytogenetic alterations of each cell line.
**Table S2.** Cell adhesion assay substrates and incubation times.

## Data Availability

The datasets used and/or analyzed during the current study are available from the corresponding author on reasonable request.
