# Supplementary material for: Exploring the role of cyclin D1 in the pathogenesis of multiple myeloma beyond cell cycle regulation
Source: Mol Oncol. 2025 Jul 17;19(11):3175–92. doi: 10.1002/1878-0261.70085 (PMC12591315; doi:10.1002/1878-0261.70085)
Supplement: Supplementary file 1 — Fig. S1. Immunofluorescence of cyclins D in the U266 cell line. Fig. S2. Quantification of mRNA expression levels of CCND1 and CCND2 in control and overexpressing MM cell lines. Fig. S3. Cell proliferation analysis in multiple myeloma cell lines overexpressing cyclin D1 and cyclin D2. Fig. S4. Cell cycle stage analysis in cyclin D1‐ and cyclin D2‐overexpressing cell lines. Fig. S5. Differential gene expression analysis. Fig. S6. Validation of the expression of genes involved in the cell adhesion molecule binding pathway. Fig. S7. Quantification of STAT1, ZO‐1, and FLNA protein levels in KMS‐28BM and KMS‐28PE cell lines. Fig. S8. Migration analysis of cyclin D1‐overexpressing MM cell lines. Fig. S9. Gating strategy and fluorescence intensity profile of several markers in a CD56‐positive and CD56‐negative MM patient. Table S1. Cytogenetic alterations of each cell line. Table S2. Cell adhesion assay substrates and incubation times. [file MOL2-19-3175-s001.pdf]

Supplementary Tables and Figures

Table S1. Cytogenetic alterations of each cell line.

Cell Line	Primary *IGH* translocation	Published chromosomal abnormalities
**KMS12-BM**	t(11;14)	69-79<3n>XX, -X, +1, +1, -4, +5, +6, +8, +9, -10, +13, +14, -15, -16, +17, -18, +19, +20, +21, -22, +4-5mar, der(1)t(1;4)(q11;q32), del(1)(p21)x2, der(1;8)(q10;q10)x2, add(4)(q32)x1-2, der(5;17)(p10;q10)x2, add(6)(q2?5), der(9)add(9)(p24)t(9;11)(q34;q13), der(9)t(9;11)(q34;q13), der(11)t(11;14)(q13;q32)x2-3, idic(?;13)(?;q32-33)x2, der(14)t(11;14)(q13;q32), der(17)t(11;17)(q21.2;q22)t(11;14)(q13;q32)x2, add(19)(q13), add(19)(q13) - related to published karyotype - **carries semi-cryptic t(11;14) with IGH-CCND1 rearrangement**
**KMS12-PE**	t(11;14)	42(40-47)<2n>X,-X, +1, +7, -14, +16, +18, -22, der(1;16)(q10;p10), der(1)(1pter->q12::18q1?-q2?1::14q21.2->q32.32::11q.13->qter)qdp(1q12.1->q12::18q1?->q21), der(3)dup(3)(q26q29)t(3;21)(q29;q11.2), del(4)(q26), der(5)t(5;17)(p10;q10), der(6)t(1;6)(q32;q22), der(7)t(7;10)(p21;p13), der(8)ins(8;14)(q24;q32.3q32.3)x2, ider(9)(q10)t(9;11)(q24;q11), der(10;16)(q10;q10), der(11)t(11;14)(q13;q32), der(11)t(9;11)(q13;q34), del(13)(q11), add(16)(p11), der(16)t(13;16)(q31;q21) der(17)t(1;17)(?;p13), add(18)(q21)x2, del(19)(q11); **carries simultaneous IGH-CCND1 and IGH-MYC rearrangements**; resembles published karyotype
**JJN3**	t(14;16)	58-67<3n>XX, +1, -2, +5, +8, +8, -9, -11, -12, -13, -15, -17, +20, add(1)(p22), der(1)t(1;?3)(q41;p26)x2, add(3)(p26), add(5)(p15)x1-2, i(5p), del(6)(q13),del(7)(q32), der(7)t(7;11)(q36;q13), add(8)(p1?)x2, t(12;19)(p13;q13), der(14)add(14)(p11)t(14;16)(q32;q23), der(14)t(14;16)(q32;q23), der(16)t(14;16)(q32;q23)x2, del(22)(q12) - **carries two copies of t(14;16) associated with c-MAF activation** - resembles published karyotype
**OPM2**	t(4:14)	78(77-82)<3n>XX-X, +1, +1, +1, +2, +4, +5, -7, +9, +11, -14, +15, +16, +18, +19, -21, +2-3mar, add(1)(p11), add(1)(q11)x2, del(1)(p11)x2, del(1)(q32), t(4;14)(p16;q32.3), der(4)t(4;14)(p16;q32.3), add(5)(q11), add(5)(q15-21), add(5)(q35), der(8)t(1;8)(?q32;q24)x1-2, add(14)(q32.1), der(22)t(1;22)(q11;p13)x2 - sideline with del(7)(p15), del(10)(q22) - resembles published karyotype - **carries cryptic t(4;14) associated with fusion of IGH with FGFR3 (MMSET)**
**U266**	t(11;14)	44(40-46)<2n>XY, -8, -10, -13, -15, +2mar, t(1;11)(p33;q13), add(3)(q27), t(4;11)(q?21;q23), add(7)(q32), add(8)(q24), add(9)(q34), add(10)(p14), add(14)(p11), add(17)(p11), add(18)(p12), der(22)t(15;22)(q21;q13) - **11q13 breakpoint recurrent in multiple myeloma**
**AMO1**	t(12;14)	
**MM1.S**	t(14;16)	
**KMS28-BM**	t(4:14)	
**KMS28-PE**	t(4:14)	

Table S2. Cell adhesion assay substrates and incubation times.

Substrate	Supplier	Final concentration	Diluent	Conditions
LDEV Geltrex	ThermoFisher	1%	RPMI1640	1 hour 37ºC
Fibronectin	Sigma Aldrich	20 µg/mL	PBS	45 min RT
Poly-L-Lysine	Sigma	50 µg/mL	H_2O	15 min RT
Collagen type-I	Corning	50 µg/mL	0.02 N acetic acid	1 hour RT

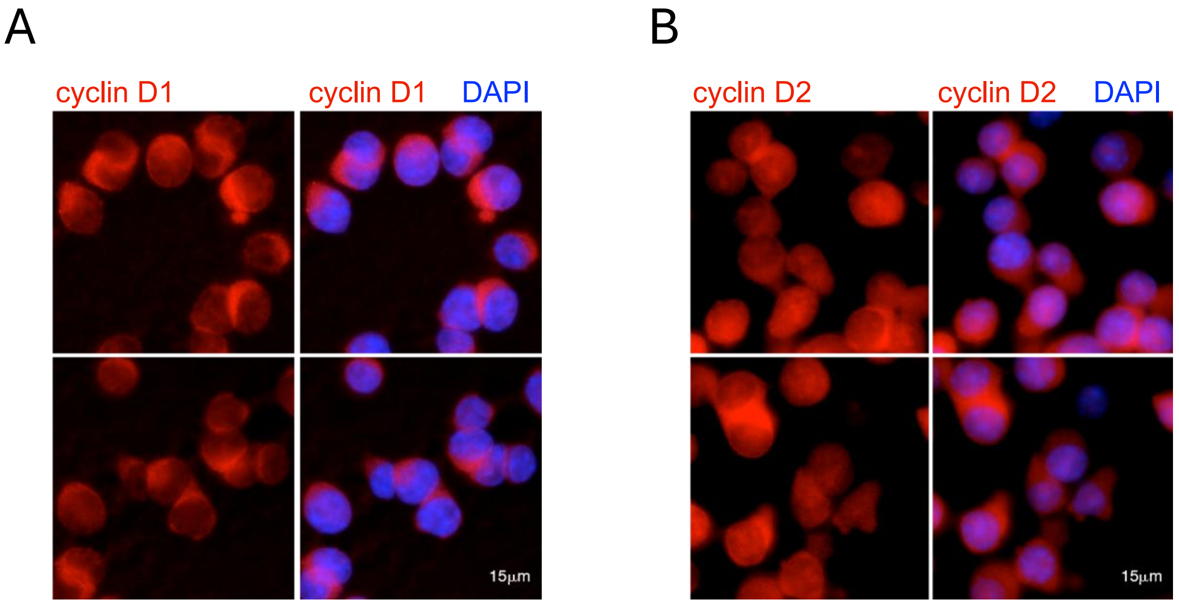

Figure S1. Immunofluorescence of cyclins D in the U266 cell line. Immunofluorescence of the **A)** endogenous cyclin D1, and **B)** endogenous cyclin D2, in the U266 cell line.

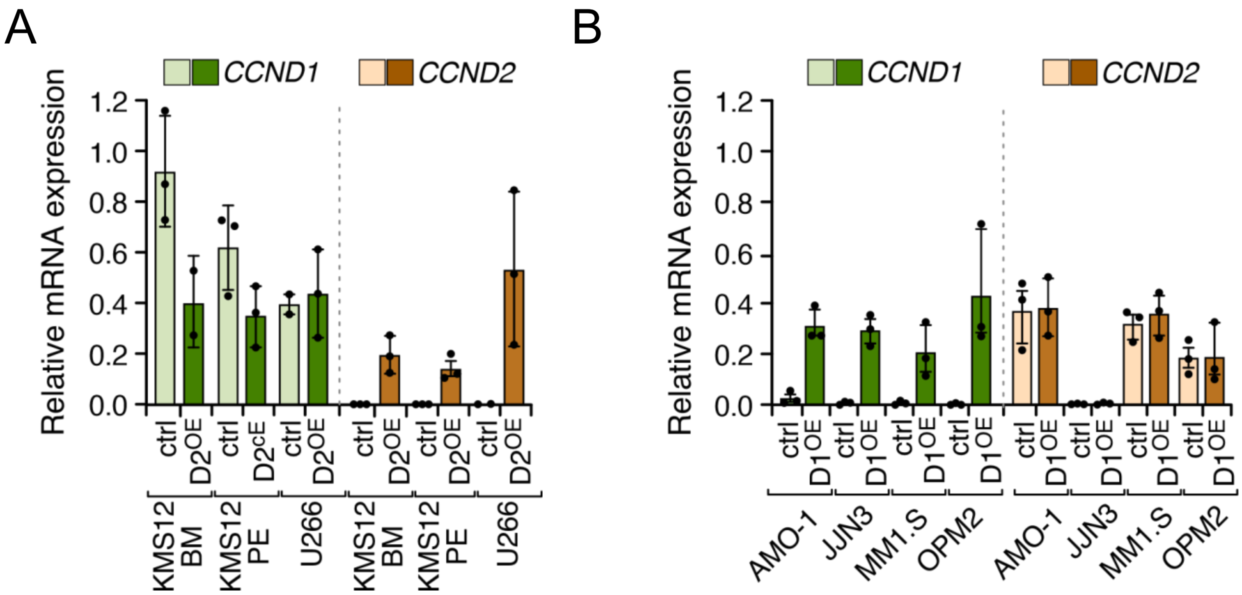

Figure S2. Quantification of mRNA expression levels of *CCND1* and *CCND2* in control and overexpressing MM cell lines. A) Quantification of mRNA expression levels of *CCND1* and *CCND2* in control and cyclin D2-overexpressing cell lines. **B)** Quantification of mRNA expression levels of *CCND1* and *CCND2* in control and cyclin D1 overexpressing cell lines. Basal levels are shown in light green (cyclin D1) and light brown (cyclin D2), while levels after overexpression of cyclin D1 and cyclin D2 are shown in dark green and dark brown, respectively. Data are from three independent experiments normalized with respect to *PGK1*. An unpaired t-test was used to establish the significance of group differences

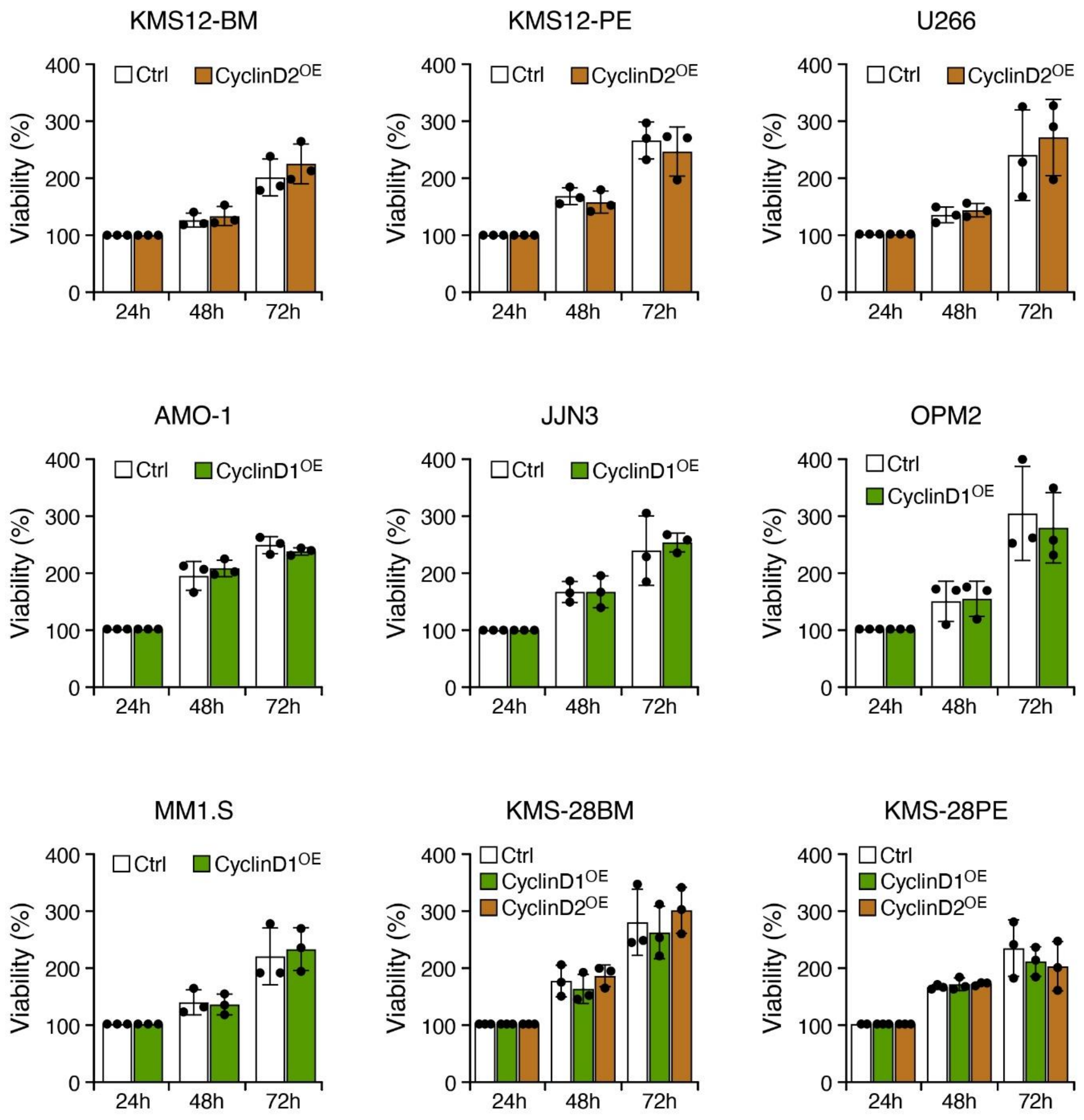

Figure S3. Cell proliferation analysis in multiple myeloma cell lines overexpressing cyclin D1 and cyclin D2. Quantification of cell proliferation at 24, 48, and 72 h using the MTT assay. MM parental cell lines are represented in white, cyclin D2-overexpressing cells in dark brown, and cyclin D1-overexpressing cells in dark green. Data are from three independent experiments. An unpaired t-test was used to establish the significance of group differences.

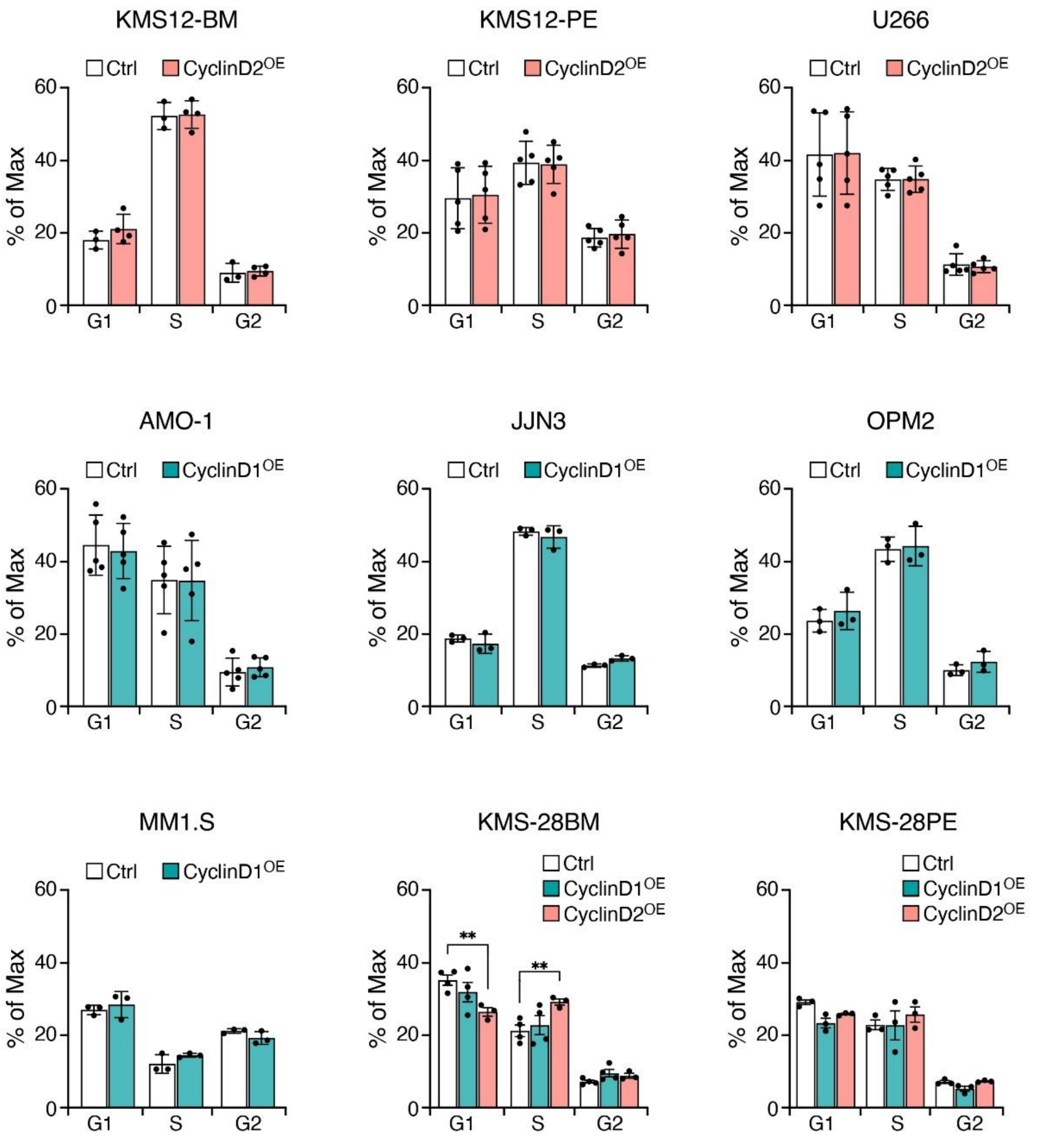

Figure S4. Cell cycle stage analysis in cyclin D1- and cyclin D2-overexpressing cell lines. Cell cycle analysis by EdU assay in MM cell lines. Parental lines are represented in white, cyclin D2-overexpressing lines in pink, and cyclin D1-overexpressing lines in turquoise. Data are from three independent experiments. An unpaired t-test was used to establish the significance of group differences (**p<0.01).

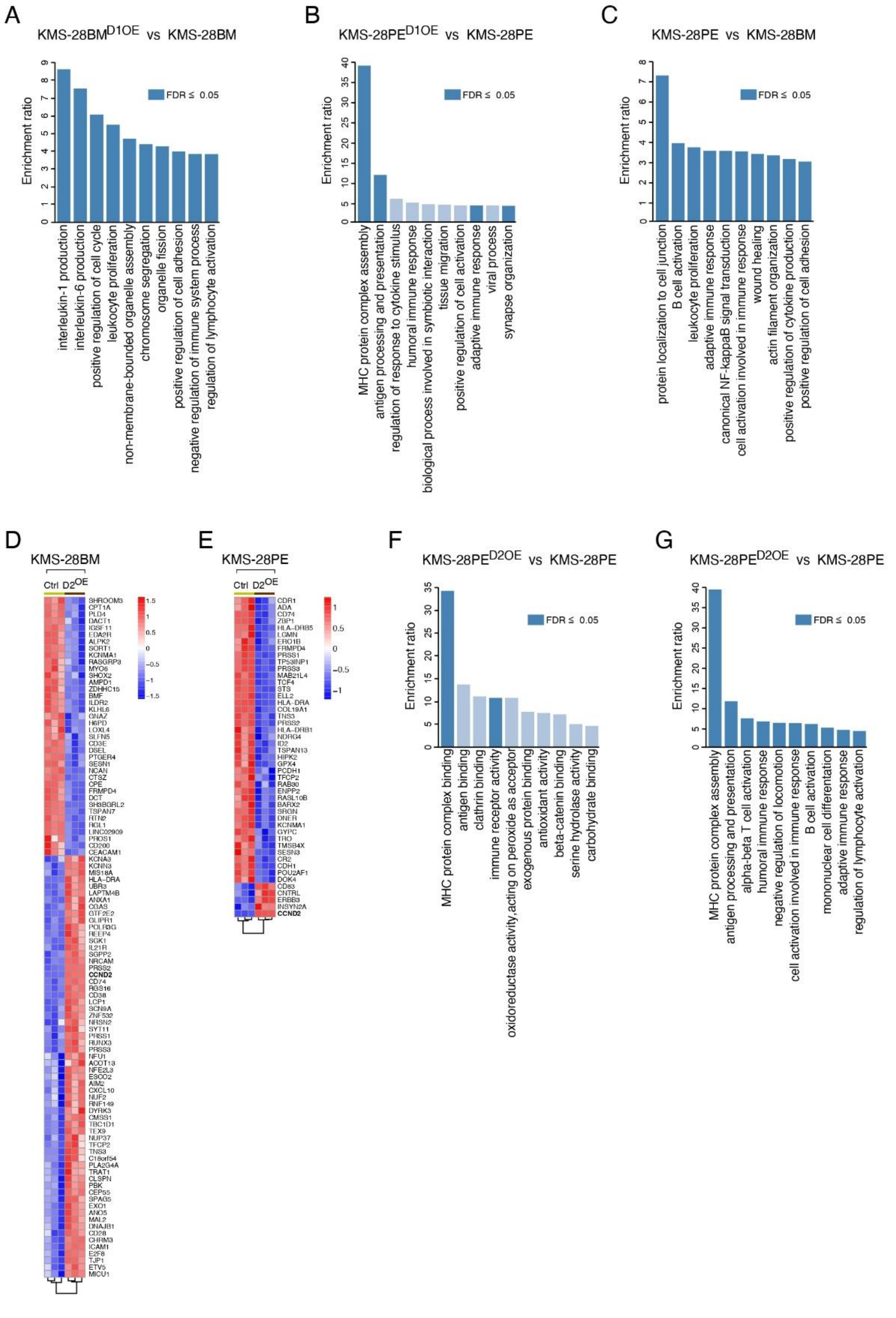

Figure S5. Differential gene expression analysis. Biological processes pathway enrichment analysis for the comparisons: **A)** KMS-28BM_D1^{OE} vs. KMS-28BM, **B)** KMS-28PE_D1^{OE} vs. KMS-28PE, and **C)** KMS-28BM vs. KMS-28PE. The Webgestalt 2024 suite was used for the enrichment analysis. Heatmap illustrating the top 100 deregulated genes in **D)** KMS-28BM_D2^{OE} compared with KMS-28BM, and **E)** KMS-28PE_D2^{OE} compared with KMS-28PE. Deregulated pathways enrichment analysis for the comparison KMS-28PE_D2^{OE} vs. KMS-28PE for: **F)** Molecular functions and **G)** Biological processes.

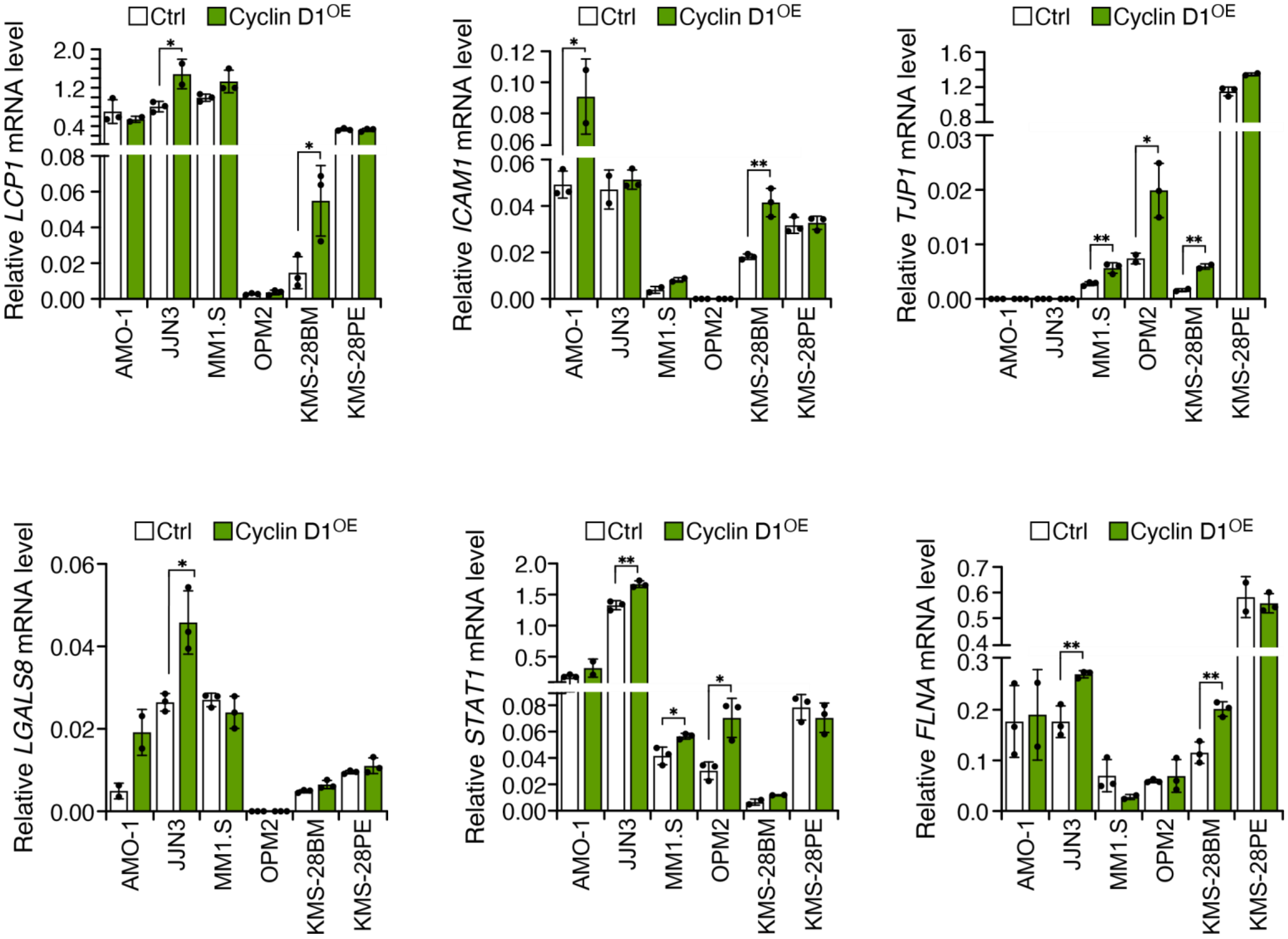

Figure S6. Validation of the expression of genes involved in the cell adhesion molecule binding pathway. Expression of *LCP1*, *ICAM1*, *TJP1*, *LGALS8*, *STAT1*, and *FLNA* genes was validated by RT-qPCR in the parental (white) and cyclin D1-overexpressing (green) cell lines. Data are from three independent experiments, and each experiment was normalized with respect to *PGK1*. An unpaired t-test was used to establish the significance of group differences (*p<0.05, **p<0.01).

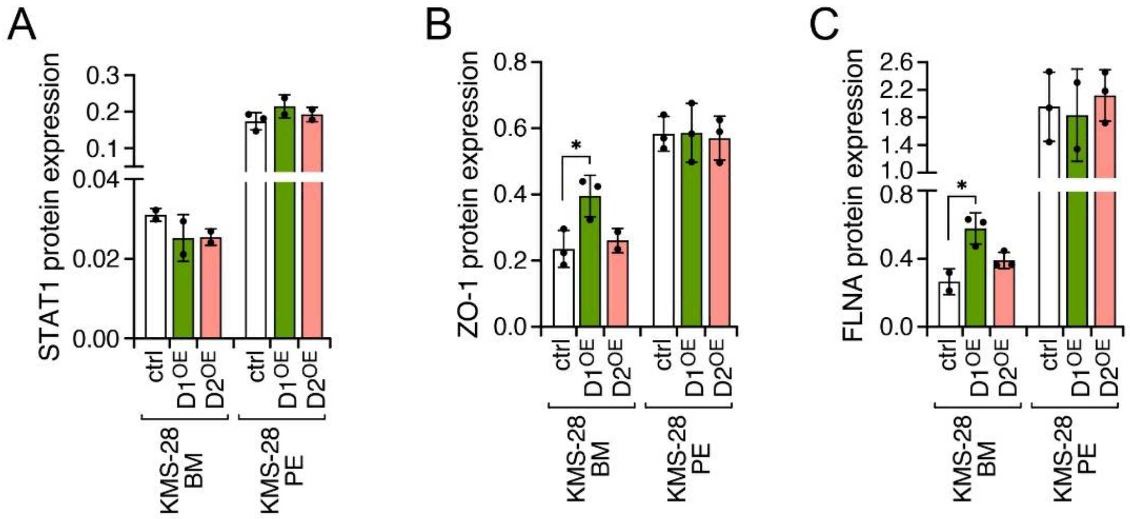

Figure S7. Quantification of STAT1, ZO-1, and FLNA protein levels in KMS-28BM and KMS-28PE cell lines. A) STAT1, **B)** ZO-1, and **C)** FLNA protein expression levels in KMS-28BM, KMS-28PE, for parental and cyclin D-overexpressing cell lines. Data are from three independent experiments. An unpaired t-test was used to establish the significance of group differences (*$p<0.05$).

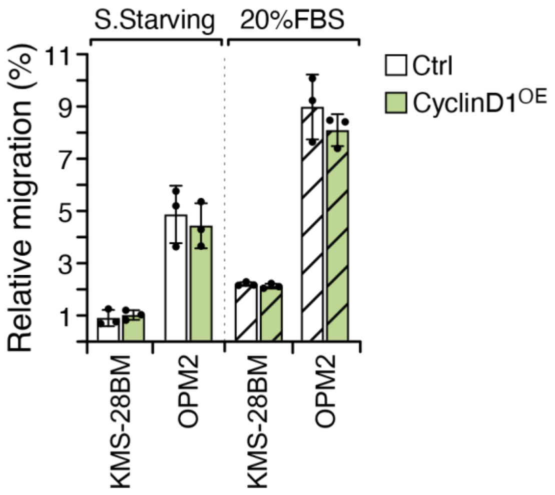

Figure S8. Migration analysis of cyclin D1-overexpressing MM cell lines. Analysis of the Transwell assay with KMS-28BM and OPM2 cell lines, including parental (white) and cyclin D1-overexpressing (green) cell lines. The bar diagram displays the relative migration under starving conditions (bars without stripes) and in the presence of 20% FBS as a chemoattractant (bars with stripes). The experiment was conducted three times independently, and an unpaired t-test was used to establish the significance of group differences.

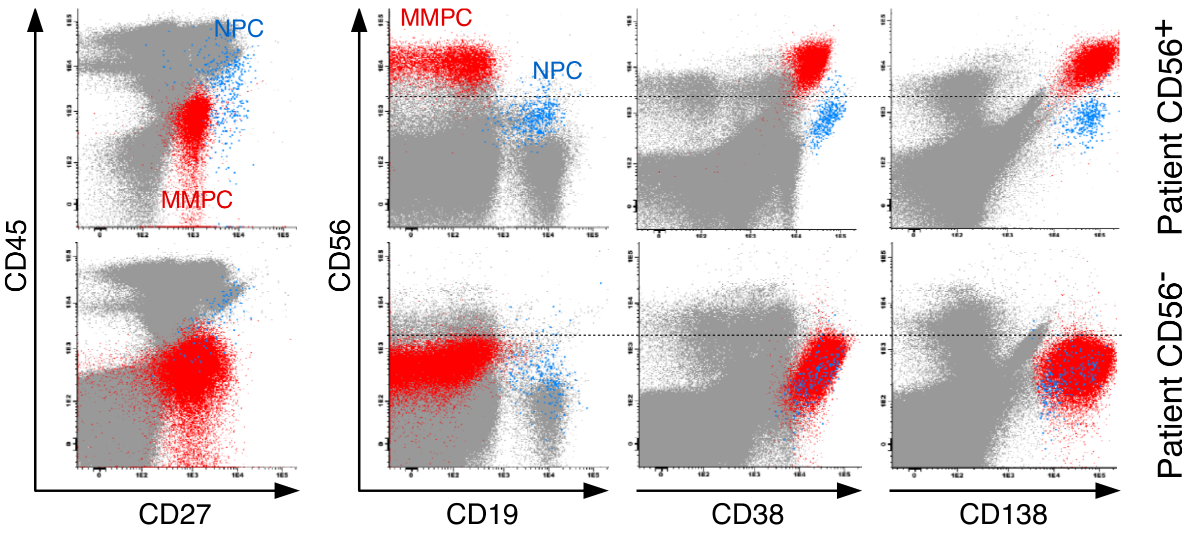

Figure S9. Gating strategy and fluorescence intensity profile of several markers in a CD56-positive and CD56-negative MM patient. Normal plasma cells (NPC) are shown in blue, multiple myeloma plasma cells (MMPC) in red, and other cell populations within each sample are shown in gray.
